# Special Issue “Breast Cancer: Molecular Pathology, Diagnosis, and Therapeutic Strategies”

**DOI:** 10.3390/ijms27010391

**Published:** 2025-12-30

**Authors:** Rodrigo Sanchez-Bayona

**Affiliations:** 1 Medical Oncology Department, Hospital Universitario 12 de Octubre, Instituto de Investigación Sanitaria Hospital 12 de Octubre (imas12), 28041 Madrid, Spain; rodrigo.sanchez@gruposolti.org; 2 SOLTI Breast Cancer Research Group, 08028 Barcelona, Spain

## 1. Introduction

Breast cancer remains one of humanity’s most significant oncological challenges, accounting for nearly 30% of all cancer diagnoses in women worldwide [1]. Despite substantial progress in diagnostic techniques and the development of targeted therapies, the aggressive and chameleonic nature of this disease—driven by extensive intratumor and intertumor spatiotemporal heterogeneity—continues to limit the effectiveness of conventional treatments. Addressing this challenge requires an ability to rethink disease mechanisms and develop novel, personalized, and theory-guided therapeutic strategies.

In this Special Issue, we present a collection of articles that explores the molecular, immunological, and ecological landscapes of breast cancer. These contributions provide critical insights into targeted treatments, immune monitoring, and the holistic principles that govern tumor progression.

## 2. Targeted Inhibition and Molecular Vulnerabilities

Triple-negative breast cancer (TNBC) represents the most aggressive subtype, frequently lacking the cellular targets found in other breast cancers and often harboring inactivating p53 mutations [2]. On et al. investigate the pharmacological inhibition of MDM2, a primary negative regulator of p53, using clinical-stage inhibitors Idasanutlin and Milademetan [Contribution 1]. Their study reveals that these inhibitors efficiently reduce TNBC cell viability and induce apoptosis regardless of p53 expression, suggesting that MDM2 is a promising therapeutic target that may function by reactivating other pathways, such as p73.

## 3. Immunological Profiles and Biomarkers

The interplay between the host immune system and tumor cells is crucial for cancer progression and therapy response [3]. Yi et al. evaluate the clinical relevance of peripheral natural killer (NK) cell activity, measured by interferon gamma (IFN-y) secretion, at the time of diagnosis [Contribution 2]. Their findings indicate that patients with stage III breast cancer exhibit significantly decreased IFN-γ secretion compared to other stages, suggesting that peripheral immune activity could serve as a non-invasive tool for pretreatment risk assessment and patient selection for immunotherapy.

Further exploring the immunological shifts induced by treatment, Roqué-Lloveras et al. examine paired tumor samples from early-stage TNBC patients before and after chemotherapy [Contribution 3]. Their research highlights a statistically significant increase in PD-L1 expression post chemotherapy, with 50% of initially negative tumors becoming PD-L1-positive. This suggests that chemotherapy can remodel the tumor microenvironment to become more targetable via immune checkpoint inhibitors, potentially opening up new windows toward targeted combined therapeutic strategies.

## 4. Innovative Diagnostic Performance

The identification of reliable imaging biomarkers is essential for assessing disease extent and selecting eligible patients for advanced treatments. Rizzo et al. provide a systematic review of the diagnostic performance of prostate-specific membrane antigen (PSMA)-targeted positron emission tomography (PET/CT) in breast cancer patients [Contribution 4]. While the diagnostic accuracy for assessing overall disease extent was modest, the high PSMA expression observed in TNBC patients suggests PSMA-PET could be a valuable tool for evaluating whether these individuals would benefit from PSMA-targeting radioligand therapy (RLT) [4].

## 5. Holistic and Ecological Approaches

Understanding breast cancer as a dynamic, evolving system is the focus of Neagu et al., who propose the “onco-breastomics” framework [Contribution 5]. This holistic approach integrates multi-omics data—genomics, transcriptomics, proteomics, and metabolomics—with ecological theories such as the River Continuum Concept and Bronfenbrenner’s ecological model to explain tumor development and progression [5]. By viewing the tumor as an “evolving dynamic ecosystem,” clinicians can better interpret complex data sets to identify non-invasive biomarkers in “river-like” liquid biopsies, aimed at reducing overtreatment and improving clinical decision-making.

The research presented in this issue underscores the biological complexity of breast cancer and highlights several promising frontiers. From targeted MDM2 inhibition and peripheral immune monitoring to the integration of ecological models for spatial biology, these studies drive us toward a future where progress is defined by systemic and personalized care. We hope these discoveries inspire foster collaboration across disciplines and ultimately contribute to a future where the clinical burden of breast cancer is greatly diminished.
